# Aortic Disease in the Young: Genetic Aneurysm Syndromes, Connective Tissue Disorders, and Familial Aortic Aneurysms and Dissections

**DOI:** 10.1155/2013/267215

**Published:** 2013-01-14

**Authors:** Marcelo Cury, Fernanda Zeidan, Armando C. Lobato

**Affiliations:** Instituto de Cirurgia Vascular e Endovascular (ICVE), São Paulo, SP, Brazil

## Abstract

There are many genetic syndromes associated with the aortic aneurysmal disease which include Marfan syndrome (MFS), Ehlers-Danlos syndrome (EDS), Loeys-Dietz syndrome (LDS), familial thoracic aortic aneurysms and dissections (TAAD), bicuspid aortic valve disease (BAV), and autosomal dominant polycystic kidney disease (ADPKD). In the absence of familial history and other clinical findings, the proportion of thoracic and abdominal aortic aneurysms and dissections resulting from a genetic predisposition is still unknown. In this study, we propose the review of the current genetic knowledge in the aortic disease, observing, in the results that the causative genes and molecular pathways involved in the pathophysiology of aortic aneurysm disease remain undiscovered and continue to be an area of intensive research.

## 1. Introduction

The aortic aneurysmal disease is defined as focal dilation of the aorta, based on its original diameter. The dilatation must be at least one and one-half times the normal diameter. Thus, generally, a segment with a diameter of greater than 3.5 cm is considered as an aortic aneurysm.

Thoracic aneurysms are those located above the diaphragm and may involve one or more aortic segments (aortic root, ascending aorta, arch, or descending aorta) and are classified accordingly. When they are located under diaphragm, aneurysms are classified as abdominal. The second ones are much more prevalent than thoracic aortic aneurysms. About 80% of aortic aneurysms occur between the renal arteries and the aortic bifurcation.

Aortic aneurysm rupture represents the 13th cause of death in the USA, accounting for nearly 15,000 of deaths annually [1]. Although, often a late onset disease, there are a significant proportion of patients with presentation at age less than 60. Cases of aortic aneurysms in children have also been reported; however, they are very rare [2]. In this group, the proportion of thoracic or abdominal aortic aneurysms and dissections resulting from a geneticpredisposition is unknown. In the absence of an identifiable genetic syndrome, familial aggregation of these aneurysms is well established. The first-degreerelatives of an individual with an aortic aneurysm have a ten to twelvefold increased risk of developing aortic aneurysms [3–5].

There are multiple genetic syndromes associated with aortic aneurysmal disease (Table 1). The Key syndromes to consider in any patient who presents at a young age an aortic aneurysm include Marfan syndrome (MFS), Ehlers-Danlos syndrome (EDS), Loeys-Dietz syndrome (LDS), familial thoracic aortic aneurysms and dissections (TAAD), bicuspid aortic valve (BAV), and autosomal dominant polycystic kidney disease (ADPKD). Less common are aortic aneurysms associated with Turner syndrome, neurofibromatosis, tuberous sclerosis, Noonan syndrome, osteogenesis imperfecta and homocystinuria.

## 2. Methods

A research was conducted of the Medline and Ovid Embase databases from 1966 to present and publications gathered using search terms of “Marfan,” “Ehlers-Danlos,” “Loeys-Dietz,” “Polycystic Kidney,” “Aortic Aneurysm,” “Genetic,” “Aortic Dissection,” “Connective Tissue,” “Familial.” Combinations of the terms above were used to generate a list of publications for this paper, as well as papers and relevant references found in the medical literature data.

## 3. Marfan Syndrome (MFS)

MFS is an autosomal dominant disorder of the connective tissue that is a relatively common disorder affecting 2 to 3 in 10,000 persons [6]. Although it produces the most significant changes in the connective fibers of musculoskeletal, cardiovascular, and ocular systems, it may also affect the pulmonary, nervous, and integumentary fibrils.

The most worrisome abnormalities associated with MFS are those affecting the cardiovascular system. Cardiovascular manifestations are mainly characterized by progressive dilatation of the aortic root, leading to aneurysm formation, which can evolute to an aortic dissection or rupture, if not well conducted and treated. Other established cardiovascular manifestations include mitral valve prolapse, dilatation of the pulmonary artery, and dilatation or dissection of the descending thoracic or abdominal aorta. It is already known that near 80% of Marfan syndrome morbidity is linked to aortic aneurysm and dissection [7].

The diagnosis of MFS is clinical. Revised clinical diagnostic criteria have been published with significant overlaps between the clinical phenotype of MFS and its connective tissue disorders [8].

Patients suspected of being MFS carriers must be submitted to a complete medical history and clinical examination emphasized on the skeletal, ocular, and cardiovascular systems. The diagnosis of these patients should be made according to Ghent criteria and requires a comprehensive clinical assessment of multiple organ systems (Table 2). Genetic testing can be useful in the diagnosis into selected cases.

Mutations in fibrillin 1 gene (*FBN1*) account for approximately 70–93% of patients who meet diagnostic criteria for MFS. *FBN1*, is a large gene (110 kb, 65 exons) with more than >600 mutations reported until this to date, located on chromosome 15.

Most recent researches associated mutations of the *TGFB2 *gene as cause of familial thoracic aortic aneurysms and dissections with mild systemic features of Marfan syndrome. Still, further work is needed [9].

## 4. Ehlers-Danlos Syndrome (EDS)

EDS is a group of connective tissue disorders characterized by articular hypermobility, skin extensibility, and tissue fragility (Table 3).

The EDS genes may be inherited via dominant autosomal, recessive autosomal, or x-linked patterns of inheritance [10].

The overall prevalence of EDS is 1 in every 10,000, depending on the type. Males and females of all racial and ethnic backgrounds have the same prevalence [10–15].

In the past, there were more than 10 recognized types of Ehlers-Danlos syndrome. In 1997, researchers proposed a simpler classification reducing the number of major types to six and gave them descriptive names: the arthrochalasia type, the classic type, the dermatosparaxis type, the hypermobility type, the kyphoscoliosis type, and the vascular type. Other forms of the condition may exist, but they have been reported only in single families or are not well characterized [10].

EDS typing is essential to evaluate the risk of aortic aneurysms. Of all the types, the *classical*, *hypermobile *and *vascular* ones are those who the aortic dilatation is found with a higher prevalence [16–21].

*Vascular EDS* (formerly EDS type IV) is an autosomal dominant disorder of type III collagen caused by mutations in *COL3A1* and occurs in 1 in 100,000 to 1 in 200,000 patients [10–12]. Key clinical features include arterial fragility or rupture, intestinal rupture, uterine rupture during pregnancy, and thin translucent skin. Other findings include, easy bruising and a characteristic facial appearance.

Minor criteria may aid in the diagnosis. If the clinical features are suggestive of vascular EDS, then the analysis of *COL3A1* can detect a mutation in 98-99% of cases [17].

Observation of structurally abnormal type III collagen from cultured fibroblasts is an alternative method for diagnosis, though it may be less sensitive in identifying vascular EDS. It is a consequence of mutations that decrease the production. Genotype-phenotype correlations are now being determined, improving the answers as far as the knowledge of the genetics studies walks, despite of the early stages of it [18].

Arterial rupture in vascular EDS is most prevalent in the 3rd-4th decade and usually involves mild-sized arteries [15], specially involving thoracic or abdominal vessels.

Frequently, descending and abdominal aorta [19]. The rupture is often spontaneous with no apparent cause. Angiography or arterial puncture is generally contraindicated, although some published cases report a successful endovascular therapies [20, 21]. Vessel surgical ligation is generally the preferred management in these cases. Any arterial clamping should be performed with extreme caution. Prophylactic surgery is not recommended. Patients can also present intracranial bleeding or stroke. Varicose veins are common in this disorder but should not be treated surgically. Bowel rupture most frequently involves the sigmoid colon.

There is no specific treatment for EDS; however, the diagnosis can help the management of the disease and in genetic counseling.

## 5. Loeys-Dietz Syndrome (LDS)

LDS is a newly described aortic aneurysm syndrome, associated with mutations of the transforming growth factor beta receptor genes 1 and 2 (*TGF*β*R1*/*TGF*β*R2*). It is characterized by premature and aggressive aneurysms and dissections, widely spaced in eyes, bifid uvula, or cleft palate, causing generalized arterial tortuosity [22]. It is inherited in a dominant autosomal manner. *TGF*β*R1*/*TGF*β*R2* gene mutations lead to an increased signaling of TGF-**β** in blood vessels resulting in overproduction of collagen, loss of elastin, and disarray of elastic fibers. There is a variable clinical expression of the disorder, and to date, clinical diagnostic criteria do not exist [23].

The emerging phenotypes of LDS are divided into two types. Type 1 LDS is a Marfan-like condition associated with severe craniofacial features (craniosynostosis, malar hypoplasia, retrognathia, cleft palate, or abnormal uvula and hypertelorism), aortic root aneurysms, aneurysm of other vessels, arterial tortuosity, arachnodactyly, pectus deformity, scoliosis, joint laxity, and developmental delay.

Type 2 LDS lacks the severe craniofacial features of type 1 LDS. It mimics vascular EDS in the association of a bifid uvula or abnormal palates aortic root aneurysm with dissection and diffuses arterial aneurysms or dissections, arterial tortuosity, and catastrophic complications of pregnancy. Patients do not have cleft palate, hypertelorism, or craniosynostosis.

Overall the median survival of LDS patients is 37 years (in contrast to 48 years for EDS and 70 years for MFS). Mortality is from thoracic or abdominal aortic dissection, rupture, or cerebral hemorrhage. Mean age for the first vascular surgical procedure is about 20 years. The majority will have aneurysms distal to aortic root. Dissection can occur without marked arterial dilatation (in contrast to MFS but similar to EDS). Perioperative vascular surgical mortality is only 1.7% (in contrast to 45% for EDS IV). These results prompt early surgical intervention in patients with LDS. Prophylactic repair of adult patients with LDS has been suggested at aortic diameters of 4.0 cm [24].

Due to the aggressive nature of LDS, genetic testing can be helpful in identifying those patients a great risk of arterial complications, who might be benefited with early surgical intervention. The sensitivity and specificity of *TGF*β*R1*/*TGF*β*R2* testing until to date is unknown. Missense mutations in the serine threonine kinase domain of *TGF*β*R1*/*TGF*β*R2* appear to account for the majority of mutations leading to LDS. There is a reducing penetrance and variable expressivity of them in LDS. Family members testing positive for a *TGFBR* mutation suggest a higher risk, but not predictive of LDS. Due to the limited number of patients reported to date, the full spectrum of patients with LDS is likely to evolve as additional patients are identified.

## 6. Familial Thoracic Aortic Aneurysms and Dissections (TAADs)

Familial thoracic aortic aneurysm disorder (TAAD) is mostly associated to ascending aorta aneurysm and dissection [25]. Familial aneurysms have been known to occur in described genetic syndromes; however, the genetic basis of nonsyndromic familial aortic aneurysms has only been recently described. These cases demonstrate a familial aggregation of aortic aneurysms. It typically presents itself at a mean age of ten years younger than nonfamilial cases (56 years versus 66 years) [26].

Currently, three genes and two loci were identified to be associated with familial TAAD. The first locus to be mapped for familial TAAD was the *TAAD1* locus at 5q13-14 [26]. The causative gene at this locus is currently unknown. The second locus to be mapped was the *FAA1* locus at 11q23-24, causing more diffuse vascular disease including both thoracic and abdominal aortic aneurysms [27].

The *TAAD2* locus was mapped in 3p24-25, and it is now known that the mutant gene at this locus is *TGF*β*R2* [28]. In individuals with *TGF*β*R2* mutations, dissection of the aorta may occur before the aorta enlarges to 5.0 cm. The majority of individuals with TAAD resulting from *TGF*β*R2* mutations, present initially aortic disease; however, the increased risk for aneurysms and dissections of other vessels includes cerebral aneurysms. The same mutations in *TGF*β*R2* have been identified in families with LDS and TAAD, indicating a broad clinical spectrum associated with the same mutations.

The second gene discovered was *MYH11.* Mutations in *MYH11* are associated with familial TAAD as well as patent ductus arteriosus (PDA) [29]. Human *MYH11* gene mutations provide the first example of direct changes in a contractile protein produced specifically in smooth muscle cells, leading to an inherited arterial disease.

Recently, a fourth gene was isolated and identified as causing familial TAAD. Missense mutations in *ACTA2 *were found to be responsible for 14% of familial TAAD cases [30]. Like *MYH11*, *ACTA2* is also involved in smooth muscle contraction. Aortic pathology from individuals with *MYH11* and *ACTA2* mutations, revealed evidence of vascular occlusive processes. In some families with *ACTA2* mutations, additional evidence of a vascular occlusive process was identified, by the presence of livedo reticularis.

There is also at least one additional major familial aortic aneurysm predisposition locus that remains to be identified.

## 7. Bicuspid Aortic Valve (BAV)

BAV is among the most common congenital heart malformations, with a prevalence of 1-2% in the general population [31]. It is associated with serious cardiovascular complications including aortic valve dysfunction, infective endocarditis, aortic dilation, aortic aneurysm, aortic dissection, coarctation of the aorta, interrupted aortic arch, cervicocephalic arterial dissection, and ductus diverticulum aneurysm.

This has led to a hypothesis of a common underlying developmental defect involving the aortic valve and the arterial wall [32]. Up to 50–70% of such patients with aortic valve dysfunction have evidence of aortic dilation and typically involves the aortic root and ascending aorta, whereas it is not present in the descending and abdominal aorta.

Root dilatation is mostly observed in younger men with BAV and is unrelated in the presence or severity of any aortic valve stenosis [33]. It is widely known that patients are not protected from subsequent aneurysm formation by aortic valve replacement (AVR), such as implicating inherent abnormalities of the aortic media [34].

Histopathological changes in the ascending aortas of patients with BAVs, including cystic medial necrosis, elastic fiber fragmentation, loss of smooth muscle cells, and changes in their orientation, have been demonstrated [35]. Analysis of the aortic media has revealed less elastic tissue and abnormalities of elastic lamellae in patients with BAV compared to patients with a tricuspid aortic valve [36].

Heritability studies indicate that BAV determination is almost entirely genetic, yet no single BAV gene has been identified. However, linkage has been established to chromosomes 18q, 5q, and 13q indicating these regions likely contain genes whose mutation results in BAV [37].

## 8. Autosomal Dominant Polycystic Kidney Disease (ADPKD)

ADPKD is one of the most common genetic disorder, with an estimated prevalence of 1 in 1,000 [37], characterized by progressive cyst development and bilaterally enlarged polycystic kidneys caused by mutations *PKD1* (85% of cases) or *PKD2* (15% of cases) [38]. Noncystic abnormalities include intracranial aneurysms and dolichoectasias, dilatation of the aortic root and dissection of the thoracic aorta and cervicocephalic arteries, coronary artery aneurysms, atrial septal aneurysms, mitral valve prolapse, and abdominal wall hernias [39, 40].

Cases of aortic dissection in patients with ADPKD demonstrate pathological findings of aortic medial cystic myxoid degeneration and support the central role of primary collagen defect in the pathogenesis of aortic dissection in ADPKD patients [41]. There have been also reported case of ADPKD associated with MFS in an adult [42]. Some cases of patients with ADPKD and abdominal aortic aneurysm (AAA) were related, but there is no definite evidence that the incidence of AAA is affected by ADPKD [43]. Overall ADPKD should be considered a connective tissue disorder regarding similarities to MFS and EDS.

*PKD1* and *PKD2* are the genes who code the proteins polycystin-1 (PC1) and polycystin-2 (PC2). PC2 forms a nonselective cation channel calcium ion permeable, and PC1 activates and stabilizes that channel. Both are detected in vascular smooth muscle cells (VSMCs) and endothelial cells of all major vessels, including the aorta and intracranial arteries.

The polycystins may be seen as cell-adhesion receptor complexes that link ubiquitous extracellular matrix components to the cell cytoskeleton. Pathologic mutations of *PKD1* and *PKD2* may disrupt calcium homeostasis and consequently affect transcription of the genes involved in blood vessel structural integrity [44, 45]. Mutations in *PKD1* and *PKD2* can cause an increase in both VSMC proliferation and apoptosis. Mouse embryos homozygous for a mutant *PKD1* or a *PKD2* null allele exhibit a lethal phenotype characterized by the diffuse of vascular ruptures and hemorrhage [44].

## 9. Turner Syndrome (TS)

Turner syndrome is a sex chromosome aneuploidy syndrome with the most frequent chromosome constitution being 45,X, affecting 1 in every 2000 live-born girls [46]. The cardiovascular anomalies include bicuspid aortic valve (BAV), coarctation of the aorta, hypertension, and thoracic aortic aneurysms and dissections. Most of these patients will also demonstrate abnormal aortic arch anatomy [47].

Aortic root dilation is observed in approximately 5% of Turner patients, and hence routine aortic root surveillance has been suggested. Aortic dissection in TS can occurs with and without associated BAV, as well as coarctation of the aorta or hypertension in aortic diameters of less than 5 cm. Seems to exists a generalized dilatation of major vessels in women with TS, including the aorta, brachial, and carotid arteries [46].

The exact origin of aortic defects in TS remains unknown although histopathology will often demonstrate cystic medial necrosis similar to MFS.

## 10. Neurofibromatosis (NF)

Neurofibromatosis type 1(NF1) is one of the most common genetic disorders occurring in 1 in 3,000 live births [48]. It is a dominant autosomal disease, which is characterized by café au lait spots, neurofibromas, and Lisch nodules [49].

Approximately half of the NF1 cases occur as spontaneous mutations with no family history of the disorder.

Arterial disease occurs in approximately 10% of NF1 patients [50]. Aneurysmatic arterial disease affects predominantly the renal arteries and less often the abdominal aorta (middle aortic syndrome) [51] which are the mainly manners.

Aortic disease due to adventitial compression from proliferation of Schwann cells followed by secondary changes of fibrosis and hence presents as stenotic or occlusive disease or due to direct invasion by Schwann cells with intimal thickening and destruction of the media and elastic tissue leading to aneurysm formation is also present [50–53].

## 11. Conclusion

Significant advances have improved the understanding of the association of genetics and aortic aneurismal disease. The disorders discussed above, integrate most of the proportion of the aortic disease, particularly in younger patients. Despite of all these technical improvements, the underlying genetic defects and aberrant molecular pathways remain unknown.

The isolation of new genes and their correlations with genetic syndromes do allow us not only to understand the pathophysiology involved in the genesis of the aortic aneurysm, but also pave the development of better clinical and surgical proposed treatments in affected individuals.

## Figures and Tables

**Table 1:** Aneurysm syndromes.

Genetic aneurysm syndromes		
Marfan syndrome (MFS)	FBN1	Dilatation and aneurysm of the aortic root, dilatation of the pulmonary artery, and dilatation or dissection of the descending thoracic or abdominal aorta

Ehlers-Danlos syndrome (EDS)	*COL5A1, COL5A2, and COL3A1 *	Arterial mid-sized rupture, specially involving thoracic or abdominal vessels. Frequently descending and abdominal aorta [18]

Loeys-Dietz syndrome (LDS)	*TGFBR1 and TGFBR2 *	Premature and aggressive aneurysms and dissections. Aneurysms distal to the aortic root

Familial aortic aneurysm and/or dissection syndromes (FAAD)	*TGFBR2, MYH11, and ACTA2 *	Ascending aorta aneurysm and dissection

Bicuspid aortic valve (BAV)	Unknown	Aortic dilation typically involves the aortic root and ascending aorta whereas it is not present in the descending and abdominal aorta

Autosomal dominant polycystic kidney disease (ADPKD)	*PKD1 and PKD2 *	Dilatation of the aortic root and dissection of the thoracic aorta

Turners	45X	Thoracic aortic aneurysms and dissections

Neurofibromatosis	NF1	Aneurysmal arterial disease affecting the abdominal aorta

**Table 2:** Major Ghent criteria used to diagnose Marfan syndrome.

System	Major criteria
Skeletal system	Pectus carinatum
	Pectus excavatum requiring surgery
	Upper to lower segment ratio <0.86 or span to height ratio >1.05
	Arachnodactyly: wrist and thumb signs Pes planus Protrusio acetabuli
	Scoliosis > 20 d or spondylolisthesis
	Reduced extensions at the elbows (<170 d)

Ocular system	Ectopia lentis (dislocated lens)

Cardiovascular system	Dilatation of the ascending aorta
	Aortic root dilatation

Dura	Lumbosacral dural ectasia

Family/genetic history	Family history
	Genetic mutations known to cause Marfan syndrome Inheritance of DNA maker haplotype linked to MFS in the family

**Table 3:** Criteria used to diagnose the vascular of EDS.

Major criteria (2 or more highly indicative)
Arterial rupture or intestinal, uterine rupture
Extensive bruising
Thin, translucent skin
Characteristic facial appearance

Minor criteria
Acrogeria
Hypermobility of small joints (fingers)
Tendon and muscle rupture
Clubfoot
Varicose veins of early onset
Arteriovenous malformation
Carotid-cavernous sinus fistula
Pneumothorax or hemothorax
Positive family history of sudden death
